# Supplementary material for: Escherichia coli cells evade inducible parE toxin expression by reducing plasmid copy number
Source: Microbiol Spectr. 2024 May 3;12(6):e03973-23. doi: 10.1128/spectrum.03973-23 (PMC11237751; doi:10.1128/spectrum.03973-23)
Supplement: Supplemental material — Tables S1-S3; Fig. S1-S8. [file spectrum.03973-23-s0001.pdf]

Supporting Information

Escherichia coli Cells Evade Inducible ParE Toxin Expression by Reducing Plasmid Copy Number

Running title: Balancing toxicity by changing plasmid copy number

Shengfeng Ruan [1], Christina R. Bourne [1 #].

[1] Department of Chemistry and Biochemistry, University of Oklahoma, Norman, OK, USA

[#] Corresponding author. cbourne@ou.edu.

Contents:

TABLE S1. Bacterial strains and plasmids used in this study.

TABLE S2. Oligonucleotide primers used for PCR cloning, sequencing, qPCR and RT-qPCR.

TABLE S3. Bacterial whole genome sequencing results compared to the published *E. coli K-12* MG1655 sequences.

Figure S1. Impact of pMind Empty Vector on *E. coli* cell viability.

Figure S2. Complete DNA sequence of the pMindBAD::*mtparE1* plasmid with gene and primer annotations.

Figure S3. Complete DNA sequence of the pMindBAD::*Strep-mtparE2* plasmid with gene and primer annotations.

Figure S4. Supplementary expression of AraE transporter didn't rescue the lack of expression of fluorescent protein mCherry in cells previously exposed to ParE1 protein expression.

Figure S5. Quantification of plasmid copy number in surviving cells.

Figure S6. Reduction in PCN is not evident for cells that have not experienced toxic protein expression.

Figure S7. Global Alignment of protein sequences of ParE1 and ParE2 toxins.

Figure S8. Analysis of RNA I and RNA II ratios in surviving cells in relation to plasmid replication regulation.

TABLE S1

Bacterial strains and plasmids used in this study.

Strains or plasmids	Description	Source of reference
Strains		
E. coli K-12		From our laboratory
MG1655	F- lambda- *ilvG*- *rfb*-50 *rph*-1	MG1655 strain was a gift from Tyrrell Conway (Oklahoma State University)
TOP10	F- *mcrA* Δ(*mrr-hsd*RMS-*mcr*BC) Φ80*lac*ZΔM15 Δ *lac*X74 *rec*A1 *ara*D139 Δ(*araleu*)7697 *gal*U *gal*K *rps*L (StrR) *end*A1 *nup*G	Invitrogen (Cat# C404010)
Plasmids		
pMind	ColE1/pMB1/pBR322/pUC origin of replication, tetRO promoter, Neo^r Kam^r	pMind was a gift from Brian Robertson (Addgene plasmid # 24730; http://n2t.net/addgene:24730; RRID:Addgene_24730)
pHerd20T	ColE1/pMB1/pBR322/pUC origin of replication, donor for *araC*-P_{BAD} fragment, pBAD promoter, Amp^r	pHerd20T was a gift from Hongwei Yu (Marshall University)
pRK2::*araE*	Constitutive promoter expressing AraE on RK2 origin	pRK2-AraE was a gift from Brian Pfleger (Addgene plasmid # 110141 ; http://n2t.net/addgene:110141 ; RRID:Addgene_110141)
pHerd20T::*mCherry*	pHerd20T carrying the *mCherry* gene in the MCS	From our laboratory
pMind::*mtparE1*	pMind carrying the *mtparE1* gene in the MCS	This study
pMind::*Strep-mtparE2*	pMind carrying the *Strep*-tagged *mtparE2* gene in the MCS	This study
pMind::*mtparD1E1*	pMind carrying the *mtparD1E1* gene in the MCS	This study
pMind::*mtparD2E2*	pMind carrying the *mtparD2E2* gene in the MCS	This study
pMindBAD::*mtparE1*	pMind::*mtparE1* tetRO promoter replaced with a 1.216-kb fragment of *araC*-P_{BAD} from pHerd20T	This study

pMindBAD::*Strep-mtparE2*	pMind::*Strep-mtparE2* tetRO promoter replaced with a 1.216-kb fragment of *araC*-P_{BAD} from pHerd20T	This study

TABLE S2

Oligonucleotide primers used for PCR cloning, sequencing, qPCR and RT-qPCR.

Primer	Purpose	Sequence (5'-3') [a]
pMind_FWD	PCR	GATATCCTTAATTAAGTATGCATCG
pMind_REV	PCR	GGATCCTGTCAGGATTCC
parE1_FWD	PCR	gtggaatcctgacaggatccGTGAGTAGCCGATACCTTC
parE1_REV	PCR	catacttaattaaggatatcTCAGAGGTTCCGGTCGAC
parD1E1_FWD	PCR	gtggaatcctgacaggatccGTGAGTAGCCGATACCTTC
parD1E1_REV	PCR	catacttaattaaggatatcTCAGAGGTTCCGGTCGAC
parD2E2_FWD	PCR	gtggaatcctgacaggatccGTGGTGGTCAACCGGGCA
parD2E2_REV	PCR	catacttaattaaggatatcTCACTCGAAGGTGCGGCC
parE2+Strep_FWD	PCR	gcagtttgaaaaaATGACGCGCAGGCTGCGC
parE2+Strep_REV	PCR	ggatggctccacatGGATCCTGTCAGGATTCCACGATGAG
araC-pBAD_FWD	PCR	tacctctagaTTATGACAACTTGACGGC
araC-pBAD_REV	PCR	tccttccacgATTATTTCTAGCCCAAAAAAAC
pMind+araC-pBAD_FWD	PCR	tagaaataatCGTGGAAGGAGGAGAGGATCCATG
pMind+araC-pBAD_REV	PCR	gttgtcataaTCTAGAGGTACCGAGCTC
gapA-qPCR_FWD	qPCR and RT-qPCR	TATGACTGGTCCGTCTAAAGACAA
gapA-qPCR_REV	qPCR and RT-qPCR	GGTTTTCTGAGTAGCGGTAGTAGC
ori-qPCR_FWD	qPCR	AGGTAACTGGCTTCAGCAGAG
ori-qPCR_REV	qPCR	TGCGCCTTATCCGGTAACTATC
parE1-qPCR_FWD	qPCR	GCAGGCACATCTGGAAGAGA
parE1-qPCR_REV	qPCR	GCCAGTCACCCGATAGAACAA
parE2-qPCR_FWD	qPCR	ACGACCTATTCGAGGCGTTT
parE2-qPCR_REV	qPCR	CGTCCGATAGGCAACGTAGT
RNA-I-qPCR_FWD	RT-qPCR	AACAAAAAAACCACCGCTACCA
RNA-I-qPCR_REV	RT-qPCR	ACAGTATTTGGTATCTGCGCTCT
RNA-II-qPCR_FWD	RT-qPCR	ACTGAGATACCTACAGCGTGAG
RNA-II-qPCR_REV	RT-qPCR	CCCTGACGAGCATCACAAA
araE-qPCR_FWD	RT-qPCR	GCGGTCGCAGGATTGTTATTTG
araE-qPCR_REV	RT-qPCR	CCCGCCATCAGGCTGTATTTA
pMindSeq_FWD	Sequencing	GGTGAGTCATAGTTGCACTT
M13_REV	Sequencing	CAGGAAACAGCTATGAC
pBAD_FWD	Sequencing	ATGCCATAGCATTTTTATCC

[a] The overlap used for gene assembly is in lower case.

TABLE S3

Bacterial whole genome sequencing results compared to the published *E. coli K-12* MG1655 sequences.

**Potential mutations**	**R002**	**R2**	**U00096.1**	**U00096.2 and U00096.3**	**ATCC 700926**	**ATCC 47076**
23S ribosomal RNA (*rrlD*)	…UCCCA AGGGUA UGGCUG UUC…	…UCCCA AGGGUA UGGCUG UUC…	…UCCCC AAGGGUA UGCUGU UC…	…UCCCA AGGG**UG AU**GCUG UUC…	…UCCCA AGGGUA UGGCUG UUC…	…UCCCA AGGGUA UGGCUG UUC…
DNA polymerase I (polA)	**N845S**	**N845S**	N845	N845	N845	N845
Long-chain fatty acid outer membrane channel/bacteriophage T2 receptor (FadL)	S383	**Frameshifted from S383**	S383	S383	S383	S383
RNase P protein component	V93	**Frameshifted from V93**	V93	V93	V93	V93
Diguanylate cyclase (DgcJ)	**IS1 family protein InsB and represser TnpA were inserted within DgcJ**	**IS1 family protein InsB and represser TnpA were inserted within DgcJ**	Intact DgcJ	Intact DgcJ	**IS1 family protein InsB and represser TnpA were inserted within DgcJ**	Intact DgcJ
Repeat sequences between Sel1 repeat-containing protein YjcO and glutamate/aspa	**2 repeats**	**2 repeats**	3 repeats	3 repeats	3 repeats	**2 repeats**

rate : H(+) symporter						
Repeat sequences between uncharacterized YcdU and tRNA-Ser	**3 repeats**	**3 repeats**	2 repeats	2 repeats	2 repeats	**3 repeats**

Figure S1

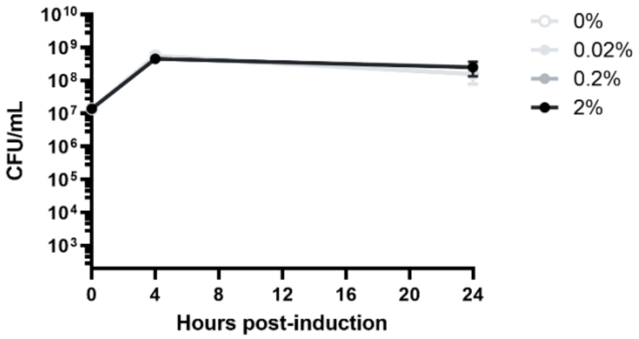

Figure S1. Impact of pMind Empty Vector on *E. coli* cell viability. *E. coli* MG1655 cells were transformed with pMind “empty” vector lacking the inserted ParE-encoding gene. Cell growths were measured with the addition of 0% to 2% arabinose. Cell viability was assessed by quantifying CFU/mL at specified time intervals (limit of detection of 200 CFU/mL). The standard error of the mean (SEM) was calculated from at least three independent experiments.

Figure S2

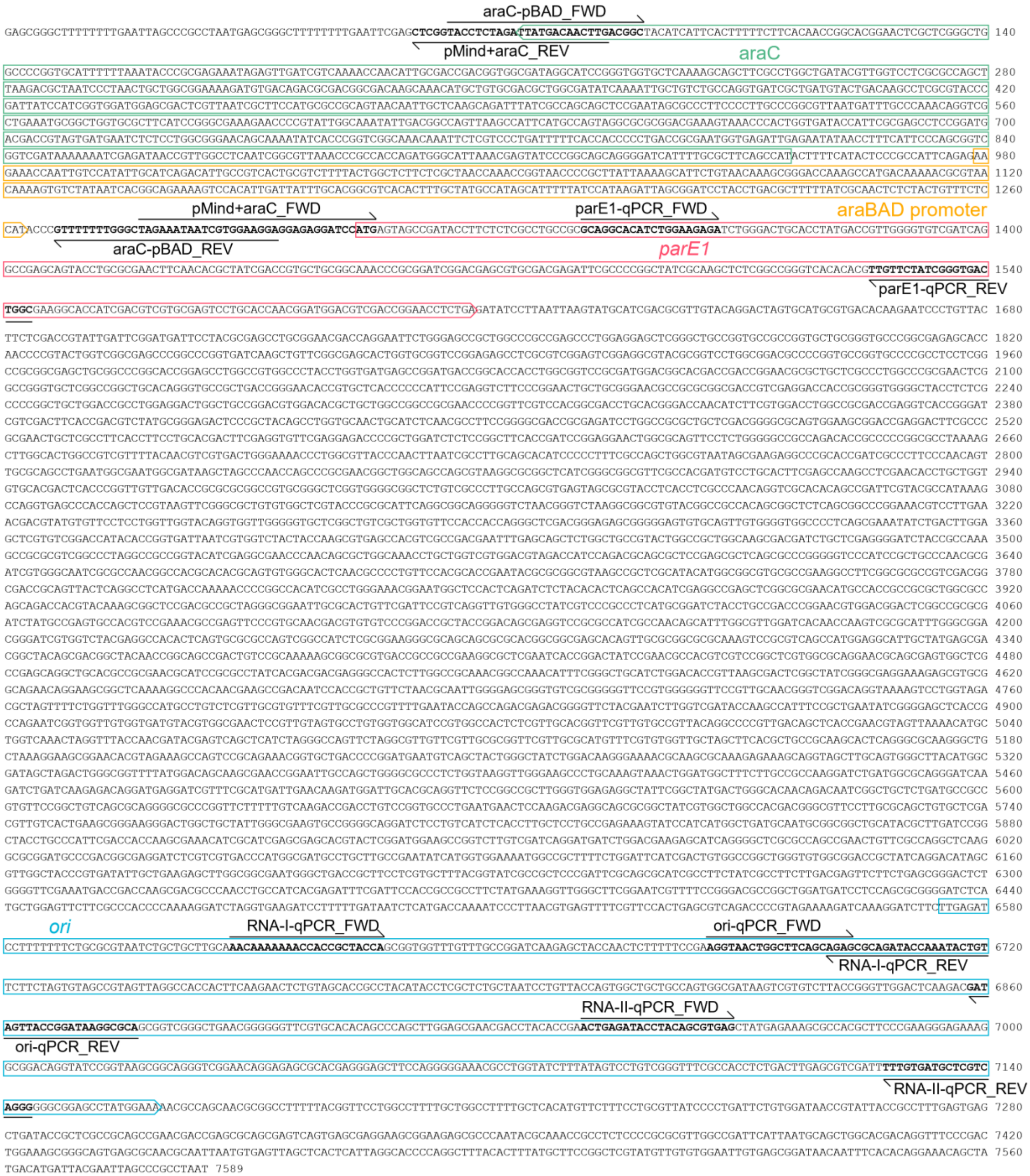

Figure S2. Complete DNA sequence of the pMindBAD::*mtparE1* plasmid with gene and primer annotations. Gene sequences are sourced from SnapGene Viewer software 7.0.3.

Figure S3

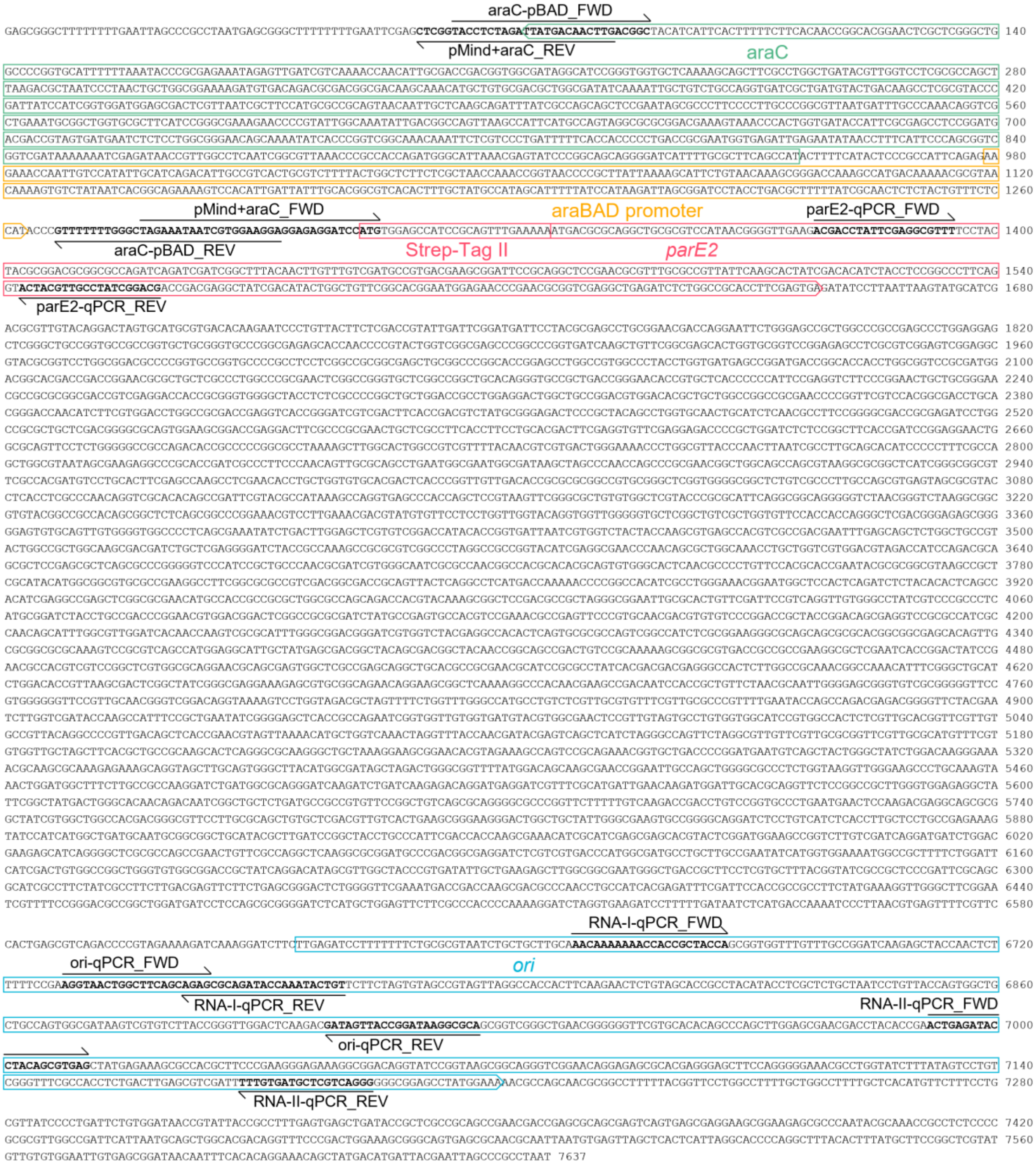

Figure S3. Complete DNA sequence of the pMindBAD::*Strep-mtparE2* plasmid with gene and primer annotations. Gene sequences are sourced from SnapGene software 7.0.3.

Figure S4

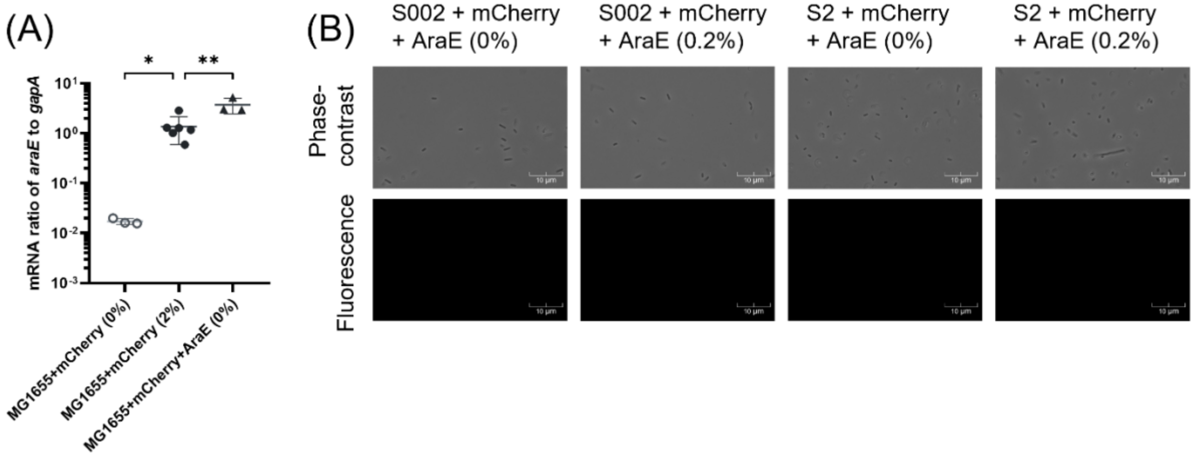

Figure S4. Supplementary expression of AraE transporter didn't rescue the lack of expression of fluorescent protein mCherry in cells previously exposed to ParE1 protein expression. (**A**) The pHerd20T reporter plasmid, carrying an arabinose-inducible fluorescent protein mCherry gene, was transformed into the "starting" *E. coli* MG1655 cells, or co-transformed with the pRK2 plasmid constitutively expressing arabinose transporter AraE into the "starting" *E. coli* MG1655 cells. Overnight cultures were induced for mCherry expression by the addition of 0% or 2% arabinose for 4-5 hr. Cells were harvested by centrifugation and RT-qPCR was employed to determine the mRNA ratio of the arabinose transporter *araE* to chromosomal *gapA* in these cells post incubation with 0% or 2% arabinose. Each measurement with standard deviation (SD) represents at least one independent experiment with each experiment containing at least 3 technical replicates. Unpaired two-tailed Student's *t*-test was performed: *, $P < 0.05$; **, $P < 0.01$. (**B**) The pHerd20T reporter plasmid, carrying an arabinose-inducible fluorescent protein mCherry gene, was co-transformed with the pRK2 plasmid constitutively expressing arabinose transporter AraE into the plasmid-cured surviving cells S002 and S2. Overnight cultures of each strain were divided into two, with one culture induced for mCherry expression by the addition of 2% arabinose for 4-5 hr, and the other one serving as a control with the addition of sterile distilled water. Fluorescence microscopy was utilized to visualize mCherry expression, with a representative image presented for each sample.

Figure S5

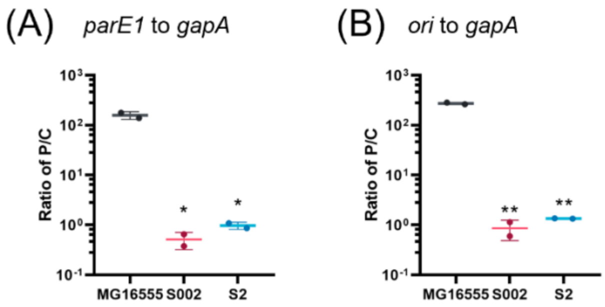

Figure S5. Quantification of plasmid copy number in surviving cells. The PCN represented by the ratio of P/C was determined by qPCR for the surviving cells S002 and S2 as well as pre-induction MG1655 cells harboring ParE1-concoding plasmid. The gapA-qPCR primer set was used for the amplification of chromosome, the parE1-qPCR (A) or ori-qPCR (B) primer set was used for the amplification of plasmid. Each measurement with standard deviation (SD) represents 2 technical replicates. The ratios of P/C of the surviving cells were compared to the ratio of P/C of the pre-induction cells and unpaired two-tailed Student's *t*-test was performed: *, $P < 0.05$, **, $P < 0.01$.

Figure S6

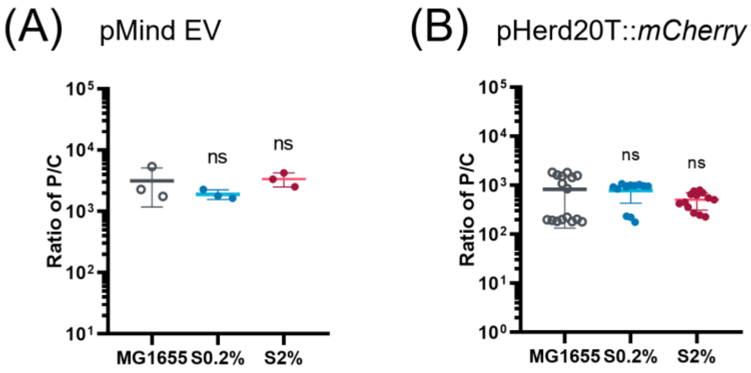

Figure S6. Reduction in PCN is not evident for cells that have not experienced toxic protein expression. The pMind “empty” vector without insertion of ParE gene (A) or the pHerd20T plasmid carrying the arabinose-inducible fluorescent protein mCherry gene (B) was transformed into the “starting” MG1655 *E. coli* cells. Following a 24-hr induction with 0.2% or 2% arabinose, cell cultures were passaged at a 1:20 dilution in fresh LB media containing the same arabinose concentration. After two 24-hr passages with arabinose inductions, at least one colony from each induction level (*e.g.*, S0.2% denotes cells survived two 24-hr passages of 0.2% induction) were isolated. The PCNs of these cells were determined by qPCR analysis. The gapA-qPCR primer set was used for the amplification of chromosome, the ori-qPCR primer set was used for the amplification of plasmid. Each measurement with SD represents 1 independent experiment, with each experiment containing three technical replicates. The ratios of P/C of surviving cells were compared to that of the pre-induction MG1655 cells and unpaired two-tailed Student’s *t*-test was performed: ns, non-significant.

Figure S7

```
# Length: 114
# Identity:      23/114 (20.2%)
# Similarity:    42/114 (36.8%)
# Gaps:          24/114 (21.1%)
# Score: 24.5
#
#
#=======================================

parE1              1 MSSRYLLSPAAQAHLEEIWDCTYDRWGVDQAEQYLRELQHA-IDRAAANP     49
                     |:.|..:....:..|.|.:.     :..|.|...:..|.:. :|  |...
parE2              1 MTRRLRVHNGVEDDLFEAFS-----YYADAAPDQIDRLYNLFVD--AVTK     43

parE1             50 RIGRACDEIRPGYRK-----LSAGSHTLFYRVTGEGTIDVVRVLHQRMD-     93
                     ||.:|.:...|.::.     |....:.:.||.|.| .||::.|.|...:
parE2             44 RIPQAPNAFAPLFKHYRHIYLRPFRYYVAYRTTDE-AIDILAVRHGMENP     92

parE1             94 --VDRNL-------     98
                       |:..:
parE2             93 NAVEAEISGRTFE*    106


#---------------------------------------
#---------------------------------------
```

Figure S7. Global Alignment of protein sequences of ParE1 and ParE2 toxins. Protein sequences are aligned using Needle (EMBOSS).

Figure S8

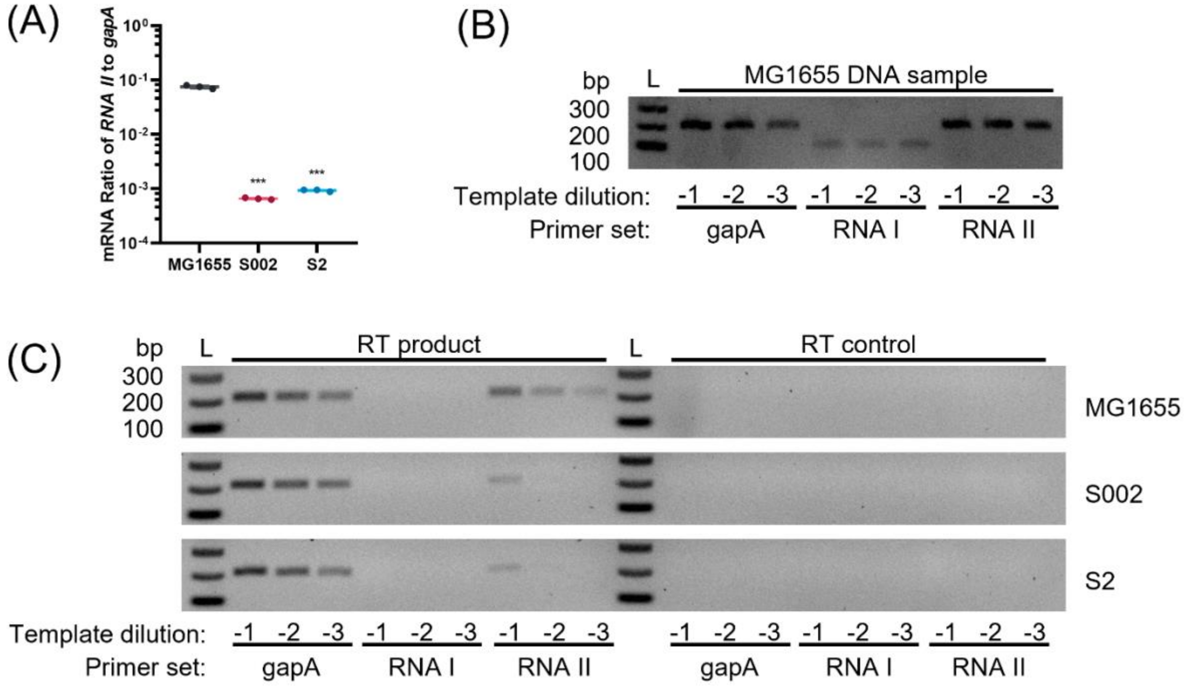

Figure S8. Analysis of RNA I and RNA II ratios in surviving cells in relation to plasmid replication regulation. The surviving cells S002 and S2 as well as the pre-induction MG1655 cells harboring pMindBAD::*mtparE1* plasmid were grown in LB media overnight without the addition of arabinose. (**A**) Cells were harvested by centrifugation and RT-qPCR was employed to determine the mRNA ratio of RNA II to chromosomal GapA. The gapA-qPCR primer set was used for the amplification of *gapA* cDNA, the RNA-II-qPCR primer set was used for the amplification of RNA II cDNA. Each measurement with standard deviation (SD) represents one experiment with 3 technical replicates. Unpaired two-tailed Student's *t*-test was performed: ***, $P < 0.001$. (**B**) The pre-induction MG1655 cells harboring pMindBAD::*mtparE1* were harvested by centrifugation and PCR was employed to amplify *gapA*, RNA I and RNA II fragments with serial dilutions of the DNA samples. The gapA-qPCR primer set was used for the amplification of *gapA* DNA, the RNA-I-qPCR and RNA-II-qPCR primer sets were used for the amplification of RNA I and RNA II complementary DNA in *ori*. PCR products were checked by 2% agarose gel electrophoresis. (**C**) PCR was employed to amplify *gapA*, RNA I and RNA II fragments with serial dilutions of the reverse transcription (RT) products from (A). The gapA-qPCR primer set was used for the amplification of *gapA* cDNA, the RNA-I-qPCR and RNA-II-qPCR primer sets were used for the amplification of RNA I and RNA II cDNA. The reverse transcriptase in RT reaction was replaced with an equal amount of water in RT control reaction.
